# Effects of W Alloying on the Lattice Distortion and Wear Behavior of Laser Cladding AlCoCrFeNiW*_x_* High-Entropy Alloy Coatings

**DOI:** 10.3390/ma14185450

**Published:** 2021-09-21

**Authors:** Tao Wu, Yunxiang Chen, Shuqin Shi, Mengting Wu, Wanyuan Gui, Yuanyuan Tan, Jiheng Li, Yuan Wu

**Affiliations:** 1 Zhejiang Institute of Mechanical & Electrical Engineering, Hangzhou 310053, China; wutao2@zime.edu.cn (T.W.); chenyunxiang@zime.edu.cn (Y.C.); shishuqin1963@126.com (S.S.); wmtwmt968@163.com (M.W.); 2 National Center for Materials Service Safety, University of Science and Technology Beijing, Beijing 100083, China; 3 State Key Laboratory of Nonlinear Mechanics, Institute of Mechanics, Chinese Academy of Sciences, Beijing 100190, China; yuanyuantan@lnm.imech.ac.cn; 4 State Key Laboratory of Advanced Metals and Materials, University of Science and Technology Beijing, Beijing 100083, China; lijh@ustb.edu.cn

**Keywords:** laser cladding, high-entropy alloy, coating, lattice distortion, high temperature wear behavior

## Abstract

Friction and wear properties of hot working die steel at above 800 °C are of particular interest for high temperature applications. Here, novel AlCoCrFeNiW*_x_* high-entropy alloy (HEA) coatings have been fabricated on the surface of hot working die steel by laser cladding. The effects of the as-prepared AlCoCrFeNiW*_x_* HEA coatings on the microstructure and high temperature friction and wear behavior of hot working die steel are investigated through scanning electron microscopy (SEM), electron probe microanalysis (EPMA), X-ray diffraction (XRD), and X-ray absorption fine structure (XAFS). Having benefited from the formation of W-rich intermetallic compounds after the addition of W elements, the high temperature wear resistance of the coatings is obviously improved, and friction coefficient shows a large fluctuation. The microstructural characteristics of the AlCoCrFeNiW*_x_* HEA coatings after the high temperature wear resistance test shows a highly favorable impact on microstructure stability and wear resistance, due to its the strong lattice distortion effect of W element on BCC solid solutions and the second phase strengthening of the W-rich intermetallic compounds. These findings may provide a method to design the high temperature wear resistant coatings.

## 1. Introduction

Currently, hot working die steel is widely used in tooling applications, including die casting, hot extrusion, hot forging, and hot stamping, due to its unique features including high strength and hardness [1,2,3,4,5]. The die surface has to withstand substantial cyclic mechanical and thermal stress, adhesion and abrasion, as well as thermal dissolution in its service process, and is often failed by repeated thermal wear and thermal fatigue [3,4,5]. Therefore, advanced surface modifications are required before the die surface can be effectively applied in practice, such as electroplating, diffusion treatment, chemical vapor deposition, cold spray, and laser cladding [6,7,8,9,10,11,12,13]. Advanced surface modification technologies can dramatically boost energy efficiency as well as improve product performance and quality [8]. Compared with other surface modification technologies, laser cladding shows great advantages in the preparation of the wear-resistance surface, as it can generate strong metallurgical bonding between cladding layers and substrate, and because of its low coating dilution rate and small thermal effect of substrate [10,11,12,13].

HEA are a class of materials that contain 5–13 principal elements in near-equiatomic proportions; they have attracted a wide range of interesting research due to their excellent mechanical properties [14]. In recent years, a variety of HEA were reported publicly in the literature [15,16,17,18,19]. Among them, AlCoCrFeNi HEA composed of a single BCC solid solution phase shows excellent corrosion resistance and has great potential for surface coating applications [13,17,19,20,21,22]. However, an insufficient high temperature wear-resistance of AlCoCrFeNi HEA greatly limits their practical applications at elevated temperatures (above 800 °C). According to the principle of dispersion strength, the strength and hardness of the simple solid solution phases could be improved further by the precipitated intermetallic compound phases. From the perspective of the dispersion strengthening mechanism, WC, TiC ceramic particles are commonly used as reinforcement phases in high-entropy alloys [10,12,23]. These granular-shaped precipitates can improve the microhardness and wear resistance of the coating. Unfortunately, it is easy to introduce cracks and defects in the laser cladding process if there are huge difference between the thermal expand coefficient of coating and that of substrate [10,12,23].

To overcome the aforementioned shortcomings, we offer an alternative approach for fabricating high temperature wear resistant HEA coatings on H13 hot working die steel. The introduction of W in the AlCoCrFeNi HEA system provides a potential solution for high temperature application because of its high melting point, low coefficient of thermal expansion, and good creep resistance [24,25]. More specifically, the introduction of W does not change the original phase composition of HEA [25]. Therefore, the performance of AlCoCrFeNi HEA is expected to be enhanced via introducing W particles. In this work, the AlCoCrFeNiW*x* (*x* = 0, 0.5 and 1) HEA coatings were fabricated by laser cladding on hot working die steel. The morphology of the microstructure and chemical composition of micro-areas were investigated in detail. The wear resistance of the coatings was tested at a high temperature, and then the wear mechanism of the worn surfaces was analyzed. We proved that the microstructure becomes more stable and enhances wear resistance of AlCoCrFeNiW*x* HEA on H13 steel by the strong lattice distortion effect of W element on BCC solid solutions and the second phase strengthening of the W-rich intermetallic compounds.

## 2. Experimental Section

### 2.1. Fabrication of AlCoCrFeNiW_x_ HEA Coatings

**Figure 1:**
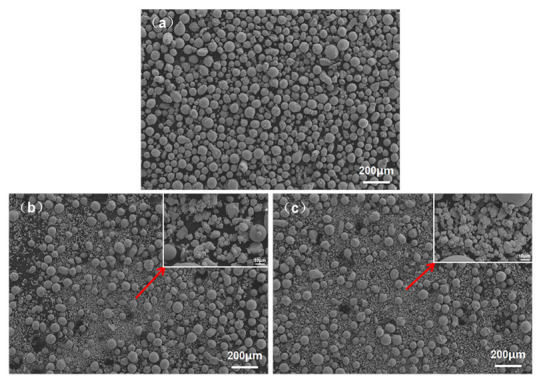
FESEEM images of the AlCoCrFeNiW*_x_* HEA powder: (**a**) *x* = 0, (**b**) *x* = 0.5, (**c**) *x* = 1.

The commercial AlCoCrFeNi HEA powder with the nominal size range of 45–105 μm and the W particles with the nominal size range of 5–20 μm (99.9% purity) were used to perform the subsequent laser cladding process, as shown in Figure 1. All powders were supplied by Jiangsu Vilory Advanced Materials Technology Co., Ltd. (Xuzhou, China). The chemical composition of AlCoCrFeNi HEA powder used in our experiments was shown in Table 1. For preparation of the AlCoCrFeNi HEA coatings, the commercial W particles powder with an atomic ratio of (0, 0.5 and 1) was mixed with AlCoCrFeNi HEA powder by ball milling (QM-3SP2 planetary, Changsha Deco Instrument Equipment Co., LTD, Changsha, China) with 300 rpm in a ball mill jar for 8 h (ball-to-powder weight ratio 3:1). Then, the mixed powders were dried under vacuum conditions (80 °C for 1.5 h) for vapor evaporation. Samples of the H13 steel were cut into pieces 10 cm × 10 cm × 0.5 cm in size. SiC paper with grit #150 was used to grind the H13 steel samples. The HEA coatings with composition of AlCoCrFeNiW*_x_* (*x* = 0, 0.5 and 1) were fabricated by laser cladding under an input laser power of 3000 W (one-layer HEA coatings with the thickness of coating being about 1 mm, and the overlapping between multiple channels about 40%). The scanning rate was adjusted into 6 mm/s, and a spot diameter was 3 mm after optimization to ensure the absence of cracks and pores in the coatings. In addition, high purity argon (99.9%) was used as a protective gas.

### 2.2. Materials Characterization and High Temperature Friction and Wear Properties

Field emission scanning electron microscopy (FESEM, Zeiss Supra 55, Carl Zeiss AG, Oberkochen, Germany) was performed for morphology observations at an acceleration voltage of 15 kV. The samples’ surface composition analysis was performed via energy dispersive spectrometry (EDS) with a take-off angle of 36.5° and live time of 2 min to further enhance the EDS precision for quantification. The phase identifications were investigated using X-ray diffractometer (XRD, PANalytical X’Pert Pro, PANalytical B.V, Alemlo, Netherlands) with Cu Kα radiation operated at a voltage of 40 kV at room temperature, a tube current of 40 mA, and a scanning rate of 10°/min, the slit width at the X-ray emitting end is 10 mm × 10 mm and at the lead skin window of the receiver is 8 mm × 20 mm. The coating surface was also examined by using energy dispersive spectrometer (EDS, Genesis Edax, Carl Zeiss AG, Oberkochen, Germany). XAFS data were collected in transmission mode from K edges of Co, Cr, Fe, Ni and W at 4B9A stations in Beijing Synchrotron Radiation Facility (BSRF, Beijing, China). High temperature friction and wear properties of the AlCoCrFeNi HEA coatings were measured by BRUKER UMT-TRIBOLAB (Beijing, China) friction and wear test. The Si_3_N_4_ grinding head was used in the test, the loading force was 5 N, the sliding friction frequency was 5 Hz, the friction stroke was 1 cm, the test temperature was 800 °C and friction time was 60 min. The width and depth of wear marks were measured by RTEC UP (Bruker. Karlsruhe, Germany) series optical profilometer instruments.

## 3. Results and Discussion

### 3.1. Phase Composition and Microstructure Feature

**Figure 2:**
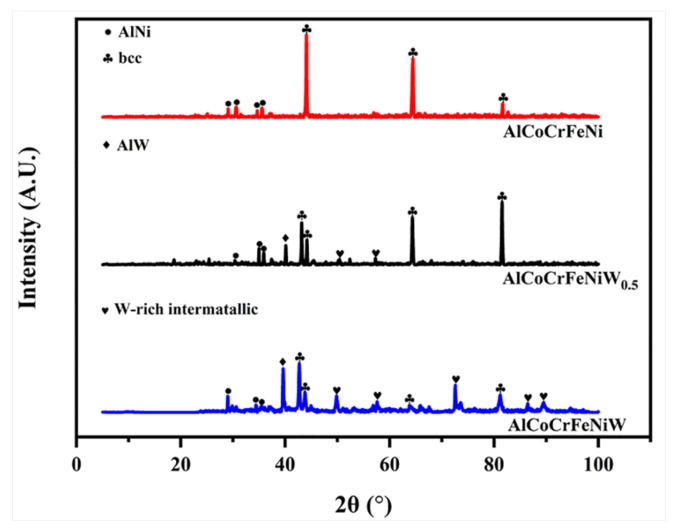
XRD patterns of AlCoCrFeNiW*_x_* (*x* = 0, 0.5 and 1) HEA coatings.

XRD patterns of AlCoCrFeNiW*_x_* (*x* = 0, 0.5 and 1) HEA coatings are displayed in Figure 2. Initially, AlNi and BCC peaks apparently occur in the diffractogram of AlCoCrFeNi HEA, which are similar to the results of previous studies [13,17]. With a 0.5 at.% W particles addition, two new phases of AlW and W-rich intermetallic compounds are detected and the AlCoCrFeNiW_0.5_ HEA are primarily composed of AlNi, AlW, BCC, and W-rich intermetallic compounds. Upon increasing the mass of the added W particles (1 at.%), the results are similar to those of the AlCoCrFeNiW*_x_* HEA with a 0.5 at.% W particles addition. Furthermore, because the atomic radius of W was larger than Co, Cr, Fe, Ni, the addition of W also caused the original lattice occupation to be distorted, which shows the shift of the peak position in the XRD patterns.

**Figure 3:**
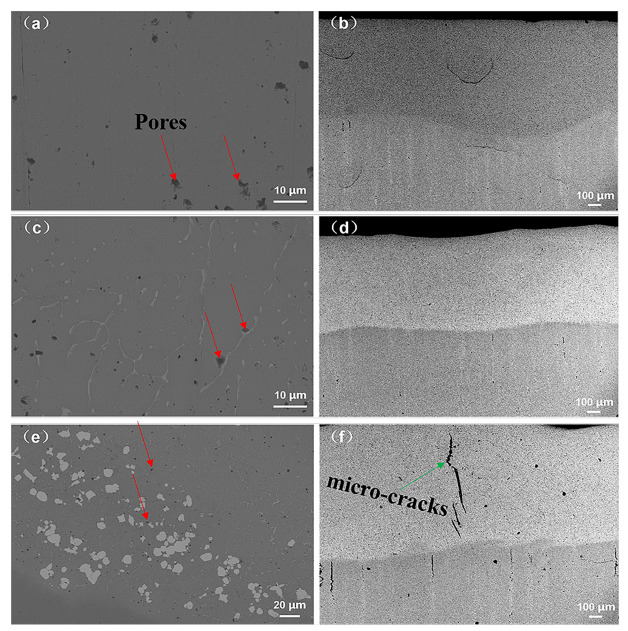
Surface and cross-sectional FESEEM images of the AlCoCrFeNiW*_x_* HEA coatings: (**a**,**b**) *x* = 0, (**c**,**d**) *x* = 0.5, (**e**,**f**) *x* = 1.

Figure 3 depicts the surface and cross-sectional morphologies of the AlCoCrFeNiW*_x_* HEA coatings. The distinct differences are observed with regard to the surface morphologies of the AlCoCrFeNiW*_x_* HEA coatings, as shown in Figure 3a,c,e. With a content of 0.5 at.% of W powder addition, some white irregular floccules appeared. As the W powder increases from 0.5 at.% to 1 at.%, the white irregular floccules microstructures transform into white suspended sphere or near sphere particulate microstructures. The cross-sectional thickness of the AlCoCrFeNiW*_x_* HEA coatings is about 850 μm, as shown in Figure 3b,d,f. Without W powder addition, there is a dense laser cladding structure with the thin 10–20 μm large micro-cracks and 1–2 μm black holes with horizontal distribution on the edge of substrate and coatings. With a content of 0.5 at.% of W powder addition, the number of micro-cracks and black holes has significantly decreased. As the W powder increases from 0.5 at.% to 1 at.%, there is a dense laser cladding structure with micro-cracks measuring hundreds of micrometers and 10–20 μm pores with vertical distribution on the edge of substrate and coatings.

**Figure 4:**
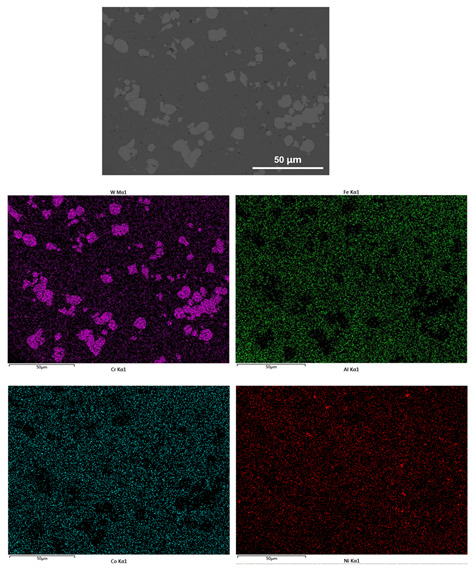

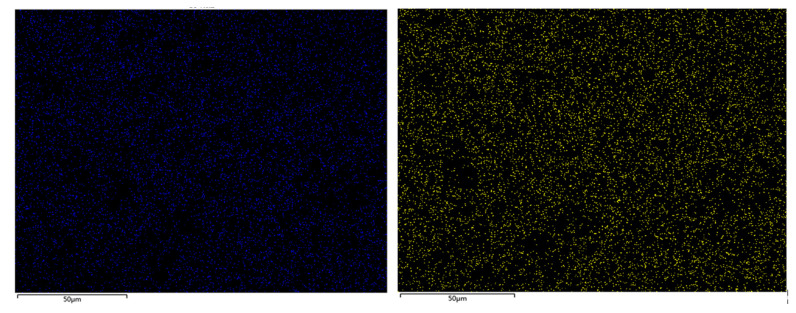
EDS element mapping of AlCoCrFeNiW HEA coatings on H13 steel.

Figure 4 shows the EDS element mapping distribution of the AlCoCrFeNiW HEA coatings, the whole coatings are distributed with W, Cr, Ni, Fe, Al, and Co elements. The EDS measurement is taken from the near upper surface of the AlCoCrFeNiW HEA coatings. Interestingly, the content of W element in white suspended sphere or near sphere particulate microstructure is much higher than other parts, suggesting that the white suspended sphere or near sphere particulate microstructure could be W-rich intermetallic compounds. In addition, the content of Al element around pores is much higher than other parts, indicating that the regions around pores were rich in Al element. The formation of the pores in coatings can be ascribed to the volatilization and oxidation of the Al element during the laser cladding process (the melting point of Al is significantly lower than that of other metals).

### 3.2. Lattice Distortion of AlCoCrFeNiW HEA Coatings Obtained by EXAFS Analysis

**Figure 5:**
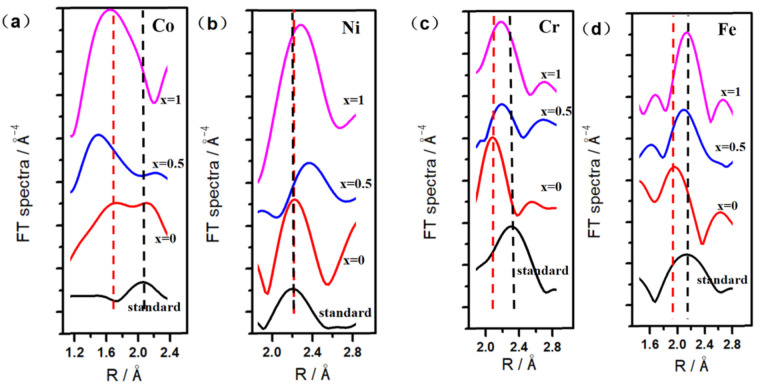
Fourier transforms spectra from EXAFS K-edges of different elements in AlCoCrFeNiW*_x_* HEA coatings: (**a**) Co, (**b**) Ni, (**c**) Cr, (**d**) Fe.

Lattice distortion results in an increase in the material internal energy and the microscopic stress, and hinders the dislocation slip deformation, making the material strength and hardness increase [14,15]. To understand the lattice distortion of AlCoCrFeNiW*_x_* HEA coatings, an X-ray absorption fine structure (EXAFS) technique [26,27] was adopted, and EXAFS measurement was taken from the near upper surface of the AlCoCrFeNiW HEA coatings. Figure 5 shows the Fourier transforms spectra from EXAFS K-edges of different elements of the standard sample and the AlCoCrFeNiW*_x_* (*x* = 0, 0.5 and 1) HEA coatings). The EXAFS data of Co, Ni, Cr and Fe K-edges were analyzed by using the Demeter software package (Univ. of Chicago) [28]. The reference spectra of their K-edges were calculated from the corresponding crystal structures by using FEFF8 code (Univ. of Washington) [29]. As shown in Figure 5a–d, the Fourier transforms spectral peak positions of all elements in AlCoCrFeNiW*_x_* HEA coatings which are significantly deviated from that of the standard sample, indicating the huge lattice distortion in the AlCoCrFeNiW*_x_* HEA coatings. It is noted that Co has negative deviation in AlCoCrFeNiW*_x_* (*x* = 0) HEA coatings, while Ni, Cr and Fe have similar positive deviation, as the W powder mass increases from 0 to 1 at.%, Co has the largest deviation or distortion, Ni has the lowest deviation. Overlapping of EXAFS peaks stems from the amplitude contribution of neighbor atoms and a photoelectron focusing effect caused by the collinear arrangement of the lattice sites [30]. These negative and positive deviations illustrate that the lattice of AlCoCrFeNiW*_x_* HEA coatings is distorted at every lattice site, since each kind of atom has some offset from the lattice sites. Deviation of peak position for Co, Ni, Cr and Fe could affect the collinear arrangement of the lattice sites, leading to further alteration of overlapping. In fact, the peak position deviation and the difference of atom spatial distribution of HEA alloys is closely related to atom size and crystal structure in pure metal and chemical bond differences in alloys. The atomic sizes of Al, Co, Cr, Fe, Ni and W are listed in Table 2. Obviously, the appreciable larger atomic size of W and Al causes the peak deviation of neighboring atoms. Reasons for peak deviations of Co, Ni, Cr and Fe after W alloying are the combined effect of appreciable atomic size, crystal structure, and chemical bonding differences. Through the high-entropy effect during alloying process, the solid solutions are crystalline, and the atoms are strongly displaced with respect to their regular location in an undistorted lattice, these results are similar to the results reported by Miracle and Senkov [31]. It was concluded that a distribution of bond length must exist in the random alloy in order for the distorted first shell of neighbors to be accommodated.

### 3.3. High Temperature Friction and Wear Properties of HEA Coatings

**Figure 6:**
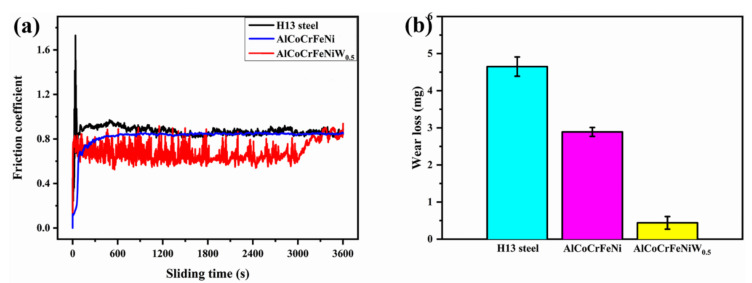
(**a**) The high temperature friction coefficient curves: H13 steel (black), AlCoCrFeNi (blue), AlCoCrFeNiW_0.5_ (red); (**b**) High temperature wear loss: H13 steel (cyan), AlCoCrFeNi (purple), AlCoCrFeNiW_0.5_ (yellow).

Figure 6 shows the influence of the W powder addition on the high temperature friction and wear properties of the AlCoCrFeNiWx coatings on H13 steel. The H13 steel exhibits a maximum friction coefficient and wear loss after 800 °C/60 min high temperature friction and wear treatment. Compared to the H13 steel, all HEA coatings exhibit smaller friction coefficient and wear loss. A minimum friction coefficient and wear loss (only 12% of H13 steel wear loss) occurs in the AlCoCrFeNiW_x_ with the addition of 0.5 at.% W. Consequently, a great improvement in the high temperature friction and wear resistance is achieved by the addition of the W powder and is likely due to the lattice distortion becoming more remarkable; the unmelted W-rich particles also improve the wear resistance as the hard phase.

**Figure 7:**
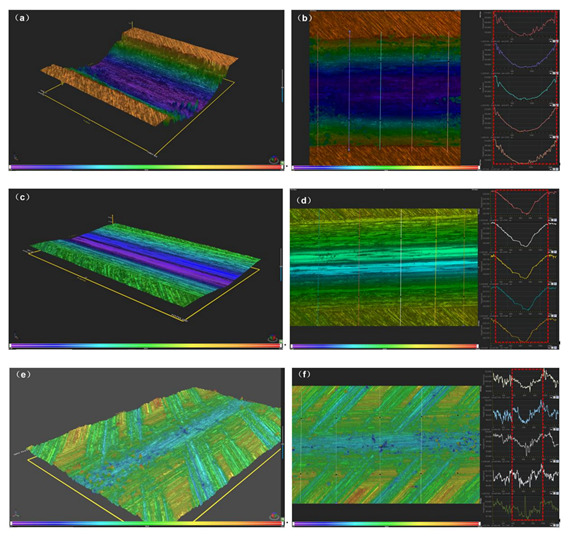
Three-dimensional morphologies and height analysis of the wear scars of samples after 800 °C/60 min friction and wear tests: (**a**,**b**) H13 steel; (**c**,**d**) AlCoCrFeNi coating; (**e**,**f**) AlCoCrFeNiW_0.5_ coating.

The addition of 0.5 at.% W powder in AlCoCrFeNi HEA system can improve its high temperature friction and wear resistance. These results are also supported by wear scars height and width of samples. The distinct differences are observed with regard to three-dimensional morphologies, as shown in Figure 7. After 800 °C/60 min high temperature friction and wear treatment, the H13 steel exhibits a maximum height and width, however the AlCoCrFeNiW_0.5_ coatings exhibit a minimum height and width. For H13 steel, there are higher and wider wear scars on its surface. Wear scars height and width of H13 steel, AlCoCrFeNi and AlCoCrFeNiW_0.5_ coatings after 800 °C/60 min high temperature friction and wear test are listed in Table 3.

**Figure 8:**
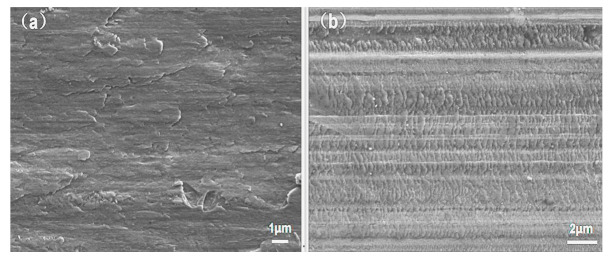
SEM images of the worn surfaces of HEA coatings after 800 °C/60 min friction and wear tests: (**a**) AlCoCrFeNi coating; (**b**) AlCoCrFeNiW_0.5_ coating.

The worn surfaces microstructure of the AlCoCrFeNi and AlCoCrFeNiW_0.5_ coatings at 800 °Care presented in Figure 8a,b. The discontinuous distribution of the adhesive layer represents the occurrence of adhesive wear during the wear process. Obvious plastic deformation occurred on both surfaces of AlCoCrFeNi and AlCoCrFeNiW_0.5_ coatings. Strong resistance of high temperature softening induced by dynamic recrystallization (involving dislocation tangles, and formation of dislocation cell structures and sub-grains) in the BCC HEA was reported recently, which indicates that dislocation tangles, and formation of dislocation cell structures and sub-grains play an importance role in the high temperature mechanical properties [32]. According to Orowan’s strengthening mechanism, lattice distortion can increase lattice stress. Lattice distortion of BCC phase for AlCoCrFeNiW_0.5_ HEA coating also plays a contributory role. Therefore, the BCC phase in the AlCoCrFeNiW_0.5_ coating has better resistance to damage under the same external forces at high temperatures. Moreover, the W-rich hard phases also contribute to improving the plastic deformation resistance of the matrix.

## 4. Conclusions

AlCoCrFeNiW*x* (*x* = 0, 0.5, 1) HEA coatings with a thickness of approximately 850 μm were successfully fabricated by laser cladding on H13 steels. Compared with AlCoCrFeNi HEA coatings with BCC phase, the addition of 0.5 at.% W powder induces the favorable formation of W-rich intermetallic compounds and BCC phase in AlCoCrFeNiW_0.5_ HEA coatings. After 800 °C/60 min friction and wear tests, the AlCoCrFeNiW_0.5_ HEA coatings exhibit a best high temperature wear consistency, due to the synergy effect of phase transformation and lattice distortion. Thus, the AlCoCrFeNiW_0.5_ HEA coatings achieve a minimum friction coefficient and wear loss (only 12% of H13 steel wear loss). These findings make noteworthy progress towards enhancing high temperature wear resistance of AlCoCrFeNi HEA coatings by laser cladding.

## Figures and Tables

**Table 1:** The chemical composition of AlCoCrFeNi HEA powder.

Element Compositions/wt.%	Element Al Co Cr Fe Ni	Tested 10.66 Bal. 20.51 22.06 22.99
Oxygen and Nitrogen content	O/ppm N/ppm	212 99
Particle size distribution	D10/µm D50/µm D90/µm	21.0 35.1 54.2

**Table 2:** Atomic size of HEA coatings’ elements (Al, Co, Cr, Fe, Ni, W).

Samples	Al	Co	Cr	Fe	Ni	W
Atomic size/Ǻ	1.43	1.25	1.30	1.26	1.24	1.41
Crystal structure		HCP				

**Table 3:** Wear scars height and width of H13 steel, AlCoCrFeNi and AlCoCrFeNiW_0.5_ coatings after 800 °C/60 min high temperature friction and wear test.

Samples	Height	Width
H13 steel	75.50 µm	1550 µm
AlCoCrFeNi coating	11.38 µm	975 µm
AlCoCrFeNiW_0.5_ coating	6.80 µm	355 µm

## Data Availability

The authors declare that the data supporting the findings of this study are available within the paper.
